# Complete genome sequence of *Enterococcus faecium* PM1 isolated from a diseased broiler breeder

**DOI:** 10.1128/mra.00830-25

**Published:** 2025-10-30

**Authors:** Vivien Zhi Ling Lim, Noele Kai Jing Ng, Guo Hao Soo, Melissa Hui Ling Ong, Yee Ting Wong, Chuan Hao Tan

**Affiliations:** 1 Kemin Industries (Asia) Pte Ltd 525362 , Singapore, Singapore; University of Pittsburgh, School of Medicine, Pittsburgh, Pennsylvania, USA

**Keywords:** *Enterococcus*, poultry, antibiotic resistance, One Health, multidrug resistance

## Abstract

We report the complete genome of *Enterococcus faecium* PM1, isolated from the gizzard of a diseased broiler breeder in a commercial farm experiencing recurrent disease outbreaks. The genome includes a 2,520,275 bp circular chromosome and four plasmids of 138,051, 15,903, 5,174, and 3,191 bp, respectively.

## ANNOUNCEMENT

*Enterococcus faecium*, part of the ESKAPE pathogens (including *Staphylococcus aureus*, *Klebsiella pneumoniae*, *Acinetobacter baumannii*, *Pseudomonas aeruginosa*, and *Enterobacter* species), is known for its use as a probiotic and emerging role as a pathogen in industry and animal husbandry (1–3). This paper reports the complete genome of *E. faecium* PM1, isolated from the gizzard of a diseased broiler breeder in a commercial farm located in Selangor, Malaysia. The farm faced recurrent disease outbreaks despite multiple antibiotic treatments.

Gizzards from a broiler breeder that died naturally during a disease outbreak were aseptically homogenized in 10 mL of sterile phosphate-buffered saline (4). A loopful of homogenate was inoculated into tryptone soy broth (TSB; Oxoid, UK) and incubated statically at 37°C for 24 h. The overnight culture was serially diluted and plated on 5% blood agar (Oxoid, UK). Colonies were purified by restreaking, from which isolate PM1 was selected. A single colony was inoculated into 10 mL of TSB with 0.6% yeast extract (Oxoid, UK) and incubated statically at 37°C for 16 h. The bacterial cells were pelleted and subsequently used for DNA extraction for short- and long-read sequencing.

The bacterial pellet was sent to an external laboratory (Axil Scientific Pte Ltd., Singapore) for short-read sequencing. Briefly, genomic DNA (gDNA) was extracted using the Quick-DNA Fungal/Bacterial Miniprep Kit (Zymo Research, USA) and quantified with a NanoDrop spectrophotometer (Thermo Fisher Scientific, USA). A paired-end DNA library was prepared using the VAHTS Universal Pro DNA Library Prep Kit for Illumina (Vazyme, China) protocol, followed by sequencing on the Illumina NovaSeq 6000 platform at 150 bp paired-end run. The quality assessment of raw Illumina short reads was conducted using FastQC (v0.11.9) (5). Reads with Phred scores <20 were removed using Trim Galore (v0.6.10) (6).

For long-read sequencing, gDNA was extracted using the QIAamp DNA Micro Kit (QIAGEN, USA) without shearing or size selection and quantified using Quant-iT dsDNA Assay Kits (Thermo Fisher Scientific, USA). A library was constructed using the Ligation Sequencing Kit V10 (SQK-LSK110, Oxford Nanopore Technologies, UK). Sequencing was performed on a MinION Mk1C device with an R9.4.1 flow cell (FLO-MIN106D, Oxford Nanopore Technologies, UK) for 24 h. Base calling was performed using Dorado (v0.4.1) (7), and reads with Phred scores <8 were removed. Read quality was assessed with NanoStat (v1.6.0) (8). Low-quality reads were filtered using Filtlong (v0.2.1) (9), removing reads <1 kbp, >500 Mbp, and the least favorable 10% of read bases.

We performed a hybrid *de novo* assembly of the short and long reads using Unicycler (v0.4.8) (10), which additionally verified the circularity of the contigs. The bacterial identity of the PM1 strain was confirmed as *E. faecium* using Kraken2 (v2.1.3) with the Standard-8 database (09 October 2023 updated; benlangmead.github.io/aws-indexes/k2) (11). The final genomes were annotated with the NCBI Prokaryotic Genome Annotation Pipeline (v6.10) (12) (Table 1). Plasmids were identified using PlasmidFinder (v2.1) (13) and PLSDB (searched by *mash dist*, v2023_11_03_v2) (14). Default parameters were used unless stated otherwise. The assembled genome includes a complete single circular chromosome and four plasmids.

**TABLE 1:** Genome features of *Enterococcus faecium* PM1

Features		Strain PM1
Illumina Sequencing	Number of reads	11,377,416
	Read length (bp)	150
Oxford Nanopore Sequencing	Number of reads	3,005,421
	Mean read length (bp)	2,720
	Total bases	8,176,427,123
	*N*_*50*_ (bp)	3,706
Final Assembly & GenBank Accession Number	Chromosome	2,520,275 bp (CP196035.1)
	Plasmids	
	pPM1_01 *(repUS15*^*a*^*)*	138,051 bp (CP196036.1)
	pPM1_02 *(rep2*^*a*^*)*	15,903 bp (CP196037.1)
	pPM1_03 *(No known*^*a*^*)*	5,174 bp (CP196038.1)
	pPM1_04 *(rep11c*^*a*^*)*	3,191 bp (CP196039.1)
	Short read coverage	636×
	Long read coverage	186×
	GC Content (%)	38.1
	Completeness (%)	98.57
Annotation Details	Genes (Total)	2,655
	CDSs (Total)	2,572
	rRNAs	18
	tRNAs	61
	ncRNAs	4

^*a*^ Predicted origin of replication.

## Data Availability

The genome has been deposited in GenBank under accession numbers CP196035 – CP196039. The associated BioProject and BioSample accession numbers are PRJNA1291100 and SAMN49947234, respectively. The corresponding raw sequencing reads have been deposited in the Sequence Read Archive under accession numbers SRR34843312 (Illumina) and SRR34844304 (Nanopore).
